# Habitually breaking habits: Agency, awareness, and decision-making in musical improvisation

**DOI:** 10.1007/s11097-024-09974-x

**Published:** 2024-03-14

**Authors:** Joshua A. Bergamin

**Affiliations:** Department of Philosophy, University of Vienna, Universitätsstrasse 7, 1010 Vienna, Austria

**Keywords:** Music, Improvisation, Pre-reflective Agency, Expertise, Participatory Sense-making, Phronesis

## Abstract

In this paper, I explore the question of agency in spontaneous action via a phenomenology of musical improvisation, drawing on fieldwork conducted with large contemporary improvising ensembles. I argue that musical improvisation is a form of ‘participatory sense-making’ in which musical decisions unfold via a feedback process with the evolving musical situation itself. I describe how musicians’ *technical* expertise is developed alongside a *responsive* expertise, and how these capacities complicate the sense in which habitual action can be viewed as pre-conscious or ‘automatic.’ Nevertheless, I shall argue that the self-awareness required for expert improvisation does not amount to highly reflective deliberation, arguing instead that the practice of musical improvisation involves an exercise of practical rationality, akin to what Aristotle called *phronēsis*. Musical decisions – as an expressive form of sense-making – are guided by feelings of ‘rightness’ that are experienced directly and intuitively, responding to the norms and reasons that are embedded in the instruments, sounds, and practices of a particular (sub)culture..

## Introduction

An intriguing paradox seems to lie at the heart of musical improvisation. On the one hand, it relies – like all musical performance – on the carefully-honed habits of musicians. Performing well, especially in an ensemble, requires seamless control of one’s instrument, a sensitivity to co-performers, and fluency in one’s chosen genre or style, all of which are developed through ongoing practice. Yet in musical *improvisation*, these well-practiced habits are also employed towards the spontaneous creation of something *new* to the practitioners themselves. To this end, a core skill of improvisation is recognising one’s own habits and patterns, so as not to respond simply ‘habitually’ during performances.

In this paper, I address this apparent paradox in the context of a phenomenology of musical improvisation, based on phenomenological fieldwork conducted with large contemporary free improvising ensembles. I argue that musical improvisation is a form of ‘participatory sense-making’ in which musical decisions unfold in a feedback process involving not only one’s fellow players, but the materials, spaces, and – most importantly – the evolving musical event itself. I describe how musicians’ *technical* expertise is developed alongside a *responsive* expertise, an exercise of practical rationality akin to what Aristotle called *phronēsis*. Both of these capacities are embodied as pre-reflective dispositions (*hexeis*) that identify and are triggered by the affordances presented by the musical event.

These capacities complicate the sense in which habitual action can be viewed as pre-conscious or ‘automatic,’ and attending to them can therefore clarify the ways in which explicit decision-making infuses pre-reflective agency. While to an important degree, musicians ‘outsource’ their choices onto the situation, the imperative to create ‘fresh’ sounds or avoid ‘clichés’ requires improvisors to attend to their spontaneous creations with mindful self-awareness. Nevertheless, I shall argue that such self-awareness does not amount to reflection, but that music-making is guided by a sense of ‘rightness’ that is experienced directly and intuitively. As a *praxis* – a holistic, lived sense-making that is expressive of its practitioners’ ‘form-of-life’ – music is articulated and interpreted pre-reflectively according to reasons and norms that are embedded in the diverse elements – instruments, sounds, and styles – that facilitate it, and that musicians come to embody via their initiation into a particular (sub)culture.

I argue this from three complementary perspectives that together offer a holistic way of understanding the forms of distributed and individual agency, knowledge, and cognition at work in musical improvisation. Firstly, in Section 2, I describe musical improvisation in the *systemic* terms of 4E-cognition, arguing that it is a form of what De Jaegher and Di Paolo (2007) call ‘participatory sense-making.’ Then, in Section 3, I discuss the *individual* perspective, drawing on phenomenological interviews with professional free improvising musicians, focusing especially on deliberation and decision-making while performing. Finally, in Section 4, I combine these accounts into a *theoretical* perspective, drawing on their common ancestry in Aristotle’s account of action in the *Nicomachean Ethics.* There, I frame the individual’s situated enactment of a systemic ‘form-of-life’ in holistic, ethical terms – focussing especially on the distinction between technical skill (*technē*) and practical ethical expertise (*phronēsis*) as situation-specific ‘modes of knowledge.’ Taken together, the three perspectives offer a mutually-reinforcing hermeneutic account that aims at articulating the interpenetration and fluid boundaries of subjective decision-making and the extended world. From this, I conclude, there is no ‘paradox’ around the role of habit in improvisation, since creative decisions result from the enactment of technical skills guided by a responsive attunement to the unique improvising situation.

## Enacting improvisation

### Improvisation in music

Before going much further, it will be useful to spend a moment looking at what improvisation *is*, and its place in music. In its broadest sense, improvisation is everywhere. It is, as Ingold and Hallam (2007, p. 1) put it, “the way we work” as social beings in a dynamic world; the way we apply our embodied experience (what Bourdieu (1977) called *habitus*) to the evolving present (cf. Lewis & Piekut, 2016, p. 9). As Peters (2009, pp. 9–10) notes, the term is used by turns pejoratively (as in ‘just playing around’) and celebratory (as the acme of creativity), where it more accurately describes an oscillating tension of adapting our practice to the circumstances at hand.

In musical practice, improvisation – defined simply as creative decisions by performers *during* the act of musicking^1^ – plays a central role. Of course, the extent to which improvisation is used, and to which musical parameters it is applied, varies widely depending on the genre or tradition in which it is employed. But throughout world musical history, improvisation has played a much larger role than a focus on western classical or modern pop music would suggest, especially in the unwritten folk and ritual traditions in which most music has been performed and experienced (Nettl, 2016). And as Benson (2003, p. 82) notes, even the most intricately-scored classical symphony contains ambiguities which performers must adapt and interpret; each performance offers unforeseen variables which shape and are shaped by the musicians’ reactions. For this reason, we speak of *interpretation*, finding one pianist’s version of Mozart’s *Sonata No. 11* preferable to another’s, even comparing recitals by the same pianist as better or worse based on subtle decisions made spontaneously during the performance.

Nevertheless, when thinking about improvisation we usually mean bigger decisions than those implied by ‘interpretation’ – not just *how* sounds are made, but *what* sounds, and *when*. But Benson’s point emphasises the sense in which improvisation describes a spectrum between structure and freedom, of which different musical traditions emphasise different elements to different degrees. ‘Improvisation’ is thus not a *particular* skill or ‘know-how’ in itself, but closer to what I have earlier called a “portfolio” of skills (Bergamin, 2017, p. 408). As Krueger (2009, p. 115) notes, music is always “irreducibly situated,” and the expertise with which a pianist improvises a cadenza to a piece by Mozart does not imply she could jump into a free jazz session in the style of Ornette Coleman.^2^ Improvisation belongs rather to a musician’s wider *praxis*, the way they combine particular skills and sensitivities (instrumental technique, a feel for rhythm, and so on) and cultural histories in performance (van der Schyff, 2019, p. 319). As I will discuss below (especially in Section 4), improvisation combines various modes of knowledge, and while the specific *technai* or skillsets that enable a particular style of improvisation will differ from context to context, their successful enactment goes together with a form of discernment that ultimately rests on a musician’s initiation into a particular musical *praxis* or ‘form-of-life.’^3^ That is to say, musical improvisation combines instrumental fluency, explicit cognitive decisions, and a deeply-felt sense of what is ‘right’ in a specific context.

### (Musical) Improvisation as participatory sense-making

If *im-pro-visation* literally describes ‘*un-fore-seen*’ spontaneous musical decisions, such decisions always happen in the context of pre-given *pro-visos*.^4^ What counts as a *proviso* is dependent on both the material but especially the socio-cultural context in which the music is performed. Hindustani classical music, for example, has quite strict rules about scales, rhythms, the musical ‘arc,’ and even the time of day certain *rāgas* should be played. But within those *provisos*, performers are free to explore and innovate in creative ways.

By contrast, the history of 20th Century jazz can be read as an ongoing loosening of *provisos*, from traditional ‘standards’ in which soloists would improvise over more-or-less fixed compositions, to more open structures and beyond, to the wild departures from traditional tonality and rhythm found in free jazz. But it’s important to note that even the ‘freest’ forms of improvisation are not without *provisos*. It is an oversimplification to aver that one can do ‘*anything*’ in free improvisation. In ‘free music’ scenes – both in jazz as well as the postwar ‘Eurological’^5^ experimental tradition – there are still *provisos* that shape and structure performances (cf. Jost, 1994). Occasionally these may take the form of explicit parameters or visual scores, but more often than not they manifest as unwritten rules or (sub)cultural norms – aesthetic values that are acquired and asserted by participating in the scene (as a performer or in audiences).

As Krueger (2011, p. 16) notes, all music is experienced “against a rich background network of social relationships and practices” or what Becker (2004, p. 71) calls a “*habitus of listening*.” These ‘forms-of-life’ influence not only *how* the music is heard but – for the performer – what kinds of responses are ‘right’ or appropriate in any particular situation. Social *provisos* therefore shape what can be played in a particular musical setting by demarcating its limits. They may be genre-specific, transmitted and enforced as cultural norms. For example, a dixieland horn player who refuses to play in the same key as the rest of the band is unlikely to be invited back to jam sessions; by contrast, a ‘reductionist’^6^ free improvisor who insists on playing bebop licks might run into similar problems in their own ‘scene.’

But *provisos* are also material, delimited both by the kinds of sounds instruments can produce, as well as how those sounds are affected by physical performance spaces (Wheeler, 2018). Again, musical culture plays a role; many experimental improvisors – through ‘preparations’ (cf. Cage, 1960) and/or idiosyncratic instrumental techniques – expand the sonic range of their instruments with respect to conventional practices. But while pushing at the limits of socio-material *provisos*, such musicians remain constrained by what is ultimately a finite set of possible sounds that can be drawn from the materials they work with, and the challenge of making those sounds ‘fit’ in different venues and constellations.

Whether as specific as a key or time signature, or as vague as a subcultural ‘sound,’ *provisos* are thus essential to the structure of improvised music. Phenomenologically, they form the *background* to improvised decision-making. *Provisos* both *enable* and *constrain* decisions, even when they are not consciously-present, and are ‘given’ insofar as they pre-exist any enactment. In a certain sense, performers “offload” or “scaffold” many of their decisions onto *provisos* (Krueger, 2019, p. 55; cf. Clark, 1997, pp. 45ff). By improvising a blues solo in A-minor, a guitarist’s options become constrained to a set of perhaps five (or six)^7^ notes, whose order will in turn be constrained by other ‘givens’ such as the physical shape and tuning of her instrument, along with cultural norms surrounding ‘root’ notes and the forms of phrases that ‘sound bluesy’ to her audience. All of these features are generally experienced ‘pre-reflectively,’ shaping the guitarist’s action without her necessarily bringing them explicitly to mind. This is partially because learning to play blues-guitar involves coming to ‘inhabit’ the instrument with fingers and ears, such that the shapes of fretboard-patterns and the sounds they elicit are felt as affordances or “lines of force” (Merleau-Ponty, 1983, p. 168). But more than that, the idea of playing (deliberately) out of key would not even ‘show up’ as a *possibility* for this guitarist, since entering into the milieu of the blues jam has *already* constrained her choices to quite specific musical parameters.

Thinking about improvised musical decisions in the context of *provisos* lends itself to a 4E (embodied, enacted, embedded, extended) cognition approach to the topic (Clark & Chalmers, 1998; Haugeland, 1998; Noë, 2004). See van der Schyff et al., 2022 for a 4E approach to musicking). What sounds are played in any given moment are not a function purely inside a musician’s head, but rely on his *habitus*, equipment, and the particular musical *situation* in which he finds himself. What each moment *affords*^8^ to any musician will be a context-dependent instantiation of all of these factors.

The unfolding temporality of music means that its affordances are also *dynamic*. Each musical decision is simultaneously a reaction to what is already happening, and an event that creates affordances for subsequent actions. This is particularly true of group improvisation, in which the actions of each performer create affordances for the others. Music-making can therefore be thought of as a form of ‘participatory sense-making’ (De Jaegher & Di Paolo, 2007). Participatory sense-making describes how agents ‘couple’ into a single dialectic process, acting on one another in a social interaction that itself emerges as a shared ‘object’ of concern (for example, a conversation) (*ibid*, p. 493).^9^ But importantly, this shared ‘object’ – while dependent on the agents from whom it emerges – has an ‘autonomy’ of its own, since through its unfolding it opens certain paths forward while closing off others, and hence develops in ways that may be independent of the intentions of any of its creators (as when a conversation drifts from one topic to another) (cf. Canonne & Garnier, 2015, p. 159).

This dialectic process around an autonomous ‘object’ finds an obvious exemplar in musical performance, where we experience.

the ‘musical object’ not as a fixed and wholly pre-given structure, but rather as an emergent phenomenon that develops through shared active involvement in the musical event; the musical object is, by this light, an ongoing open structure that *shapes and is shaped by* the sensemakers *in a circular fashion*.

(Schiavio & De Jaegher, 2017, p. 34; my emphasis).

As many improvisors have noted, even a solo improvisation involves this sense of the music as ‘autonomous’ and not entirely under the musician’s control (Nachmanovitch, 1990, p. 4; McAuliffe, 2021, p. 43; Høffding & Snekkestad, 2021, p. 168). Each moment offers new opportunities and constraints on a musician’s action, always with the imperative that each decision needs to ‘make sense’ in the context of the genre or piece. Of course – as with a conversation – there is always a possibility that it simply *won’t* make sense. But when everything is going well, a musical improvisation unfolds with the fluency of a dialogue, with the sense that the music is ‘going somewhere’ or even that it “wants” to be a certain way (Borgo, 2014).

## A phenomenology of musical improvisation

### Background and methodology

In the previous section, I described how the 4E-cognition program lends itself to understanding improvisation as a dynamic system, a form of participatory sense-making where cognitive processes extend beyond any individual agent to encompass instruments, co-performers, and of course, the unfolding music itself. This approach, grounded in the cognitive sciences, takes a somewhat abstract stance, but by no means disregards the significance of the individual as a key node in this system. Nevertheless, as Kyselo (2014, pp. 6–7) has noted, there is sometimes a tendency in enactivist thinking to downplay person-level agency, seduced perhaps by biological analogies of homeostasis that site the source of action in a mutual interaction of agent and environment. But social sense-making can be messy and disharmonious, and musical improvisation offers countless examples of misunderstandings or clashes of will, where acting ‘as one’ might not even be a shared experience (Wolf et al., 2023). While it is helpful to view the improvising ‘situation’ as a single system – thereby blurring traditional conceptions of agency, freedom, and decision-making – this raises in its turn questions about *how* decisions are enacted from the perspectives of autonomous agents as they navigate the very system they create.

Several empirical studies have investigated the interplay between individual decision-making and group agency in collective musical improvisation. For example, a series of quantitative studies by Canonne, Goupil, Saint-Germier, and colleagues (Canonne & Garnier, 2015; Goupil et al., 2020; Goupil et al., 2021; Wolf et al., 2023; Golvet et al., forthcoming; Saint-Germier et al., forthcoming) explore aspects of how and when musicians make explicit decisions. By collecting real-time signals from performing improvisors, or continuous ratings immediately afterwards, these researchers have documented how musical choices and the emergent musical forms are perceived and understood by co-performers and (initiated) listeners. Wilson and MacDonald (2016, 2017), taking a qualitative tack, approached similar questions by interviewing improvisors about the explicit decisions they made in a freshly-recorded improvisation, and about the socio-musical considerations that grounded those choices. The scholars then schematised the kinds of options that are available to improvisors (such as initiating musical material, or responding to another’s by augmenting or contrasting it (Wilson & MacDonald, 2016, p. 1035)), discussing the social qualities (a sense of trust, or the tension between predictability and surprise (Wilson & MacDonald, 2017, p. 141)) that facilitate and constrain how improvisations develop (Wilson & MacDonald, 2016, p. 1039).

Taken together, these studies suggest how emergent forms are perceived by improvisors and experienced as affordances that shape how musicians develop them, lending weight to a conception of improvising ensembles as dynamic, sense-making systems. But as Inkpin (2016, pp. 311–2) argues, such forms – as ‘scaffolded’ or externalised cognitive processes – must have “an irreducible experiential side that can become the object” of a first-person description, and that such description is necessary to complete the cognitive scientific explanation. In the case of musical improvisation, an additional motivating problem is that key phenomena – not just implicit *provisos* but also cognitive acts like decisions – may only be experienced *pre-reflectively*. That is, the forms of knowledge, perception, and agency in immersed action do not always align with straightforward conceptions of subjects acting on objects (Taylor, 2005, p. 34; cf. Merleau-Ponty, 1983, p. 168), and there remain open questions about how explicit decisive acts mesh with other factors – like musical habits and *provisos* – to facilitate and constrain those decisions (Christensen et al., 2016).

Investigating this calls for a phenomenological approach. As a methodology, phenomenology shares several key concepts with 4E-cognition accounts – such as a resistance to mind–body and mind-world dualisms, and a conception of subjectivity as situated – and, indeed, was itself influential in foundational formulations of the 4E program (Thompson, 2007; Varela et al., 1991). Nevertheless, by remaining firmly rooted in the first-person experience, it offers a distinct perspective on *how* situated cognition is enacted, and the (often implicit) *provisos* that structure it.

But a historical weakness of phenomenology is that – by dint of its focus on the first-person – it tends to be undertaken from the point-of-view of the phenomenologist himself. As Høffding (2018, pp. 13–4) notes, it is uncommon for experts in most fields to have phenomenological training (and vice versa, although artist/scholars like David Sudnow (1993) offer notable exceptions). To bridge this gap, Høffding & Martiny (2016), drawing upon work by Ravn and Hansen (2013) and Petitmengin (2006) among others, have developed a practice of ‘phenomenological interviews’ in order to gain *second*-person phenomenological access to the first-person experiences of experts. Høffding & Martiny (2016, pp. 543–4) summarise their method as comprising two ‘tiers.’ Within the first tier – an open, semi-structured interview – the researcher guides the subject to give rich descriptions of their experience, focusing attention on the ‘things themselves.’ The second tier is then a phenomenological analysis of those descriptions by the researcher, who ‘brackets’ the ‘natural attitude’ (cf. Husserl, 2013) of the informants in order to articulate the structures of the experience itself, as revealed in the subject’s descriptions. Over the course of multiple interviews, the two tiers feed back into one another, with the developing analysis informing the kind of questions that are asked, and phenomena that are targeted. The phenomenological themes are thus arrived at via “inductive” (from the ‘bottom-up’) analysis (Braun & Clarke, 2006, pp. 83–4), in contrast to the “theoretical” (‘top-down’) (*ibid*) approach taken in other phenomenological studies such as Saint-Germier et al. (forthcoming).

Høffding’s own work focuses chiefly on musical absorption in a classical string quartet, although he has also collaborated on a detailed account of a free-improvising saxophonist’s practice (Høffding & Snekkestad, 2021). Denzler and Guionnet (2020) have assembled a rich but uninterpreted collection of detailed descriptions from leading free improvisors, but there remains a gap for a more systematic and engaged phenomenology of spontaneous musical creativity. In what follows, I draw upon interviews conducted over the course of three musical ‘Labs’ with professional improvising ensembles. A total of 65 interviews (typically lasting between 30–60 min, for a total of 34.5 h) were conducted with 38 professional improvisors (of which 14 female). Each musician has extensive live performance experience and, with rare exceptions, at least ten years’ experience as a professional ensemble improvisor.^10^ The ensembles themselves specialise in various forms of jazz, free, and experimental improvisation. As is typical of the European free improvising scene, performers had diverse backgrounds, spanning from conservatory-trained musicians to self-taught artists working with homemade instruments.

Each Lab comprised a weeklong workshop and concert preparation session during which ensembles engaged in free playing as well as musical exercises aimed at topicalising specific musical parameters (such as rhythm or the effect of different spatial configurations). Musical performances were used as ‘jumping off points’ for interviews, since, as a multi-instrumentalist myself, I could closely observe individual practices and group habits, and start conversations about where musicians directed their attention, how they reacted in specific moments, what was salient to them (or not), as well as more general questions about their practice. Initial short interviews took place during rehearsal sessions and breaks, questioning musicians over actions and materials while they were still freshly in mind. These then formed the basis for longer, more in-depth interviews over the course of the Labs, which took place as semi-structured conversations exploring situated and related experiences. A key task as an interviewer was to gently lead subjects from making interpretations back to the describing the phenomena itself, using probing questions like “*how do you know when to do* x*?*”, “*what does* that *feel like?*”, “*why did you stop playing then?*” and so forth, in order to encourage musicians to articulate their experience of affordances, decisions and reactions, and socio-musical phenomena.

Combining the dialectic of the interview with musical practice is an ideal method for investigating experiential structure because, as Noë (2015, p. 29) argues, artists themselves perform a kind of philosophical research, “reorganising” and problematising everyday “lower level organised activities.” On Noë’s account, a musical *praxis* is to music as philosophy is to thinking. Musicians – especially experimental artists like the ensembles featured here – are extremely sensitive to the elements of their practice, and the phenomenologist’s task is to translate and interpret these artistic researchers’ knowledge into philosophical terms. Interpretation here, following the ‘two-tier’ methodology, includes bracketing the ‘natural attitude’ of informants’ own interpretations to get closer to unarticulated subtleties in their experience. For example, many musicians would conclude that an improvised decision was ‘just intuitive.’ And while this is an accurate description of how an act *feels* and is accomplished in the moment, we shall see in the next subsection that – upon probing and interpretation – the experience can be further unpacked to reveal the habituated pre-reflective attention that underlies a rational but non-deliberative mode of cognition.

Interpretation was iterative across individuals, ensembles, and Labs. Topics that arose in rehearsal informed in-depth interviews, which in turn informed subsequent interviews, and were also revisited in rehearsals. Between Labs – during and following transcription – interview texts were tagged and inductively thematised around key phenomenological topics that emerged across subjects (cf. Braun & Clarke, 2006), including *thinking*, *reacting*, *impulses*, *flow*, *monitoring* and *split attention*, which I discuss in this article. These themes were arrived at via ‘second-tier’ analysis of musicians’ descriptions, itself an iterative process that developed concepts as common experiences arose from one Lab to the next. The quotes presented below have been selected as representative, and while for the purposes of this essay they are primarily illustrative, their relevance is supported by the immersive Lab context. Working closely with the informants through an intensive rehearsal process, including countless informal conversations during breaks and downtime, assured me that I agreed with the musicians on the phenomena in question. As an active musician myself, I was even invited by one of the ensembles to join them musically (including in a public concert), allowing me to ‘test’ my understanding ‘from within’ in an ecological setting post-interview, thus giving me a claim to what Collins (2010, p. 123) calls “interactional expertise.” Nevertheless, I have attempted to ‘bracket’ my own experience from the descriptions that follow, which are distillations of the informants’ own accounts.

#### Results: A caveat

As noted above, music is always “irreducibly situated” (Krueger, 2009, p. 115) and as such there follows an important caveat that this section can offer only *a* phenomenology of musical improvisation, rather than *the* definitive account. The task of this paper is to identify phenomenal categories present in the observed musical situations, but although my research centres on one particular (albeit transnational) free improvising ‘scene,’ we will see reason to believe that the phenomena, skills, and forms of attention I articulate are present – at least in broad-brushstrokes – in other improvising practices, and could be developed with reference to other styles.

Most music performed in the Labs could be described as ‘non-idiomatic’ free improvisation (Bailey, 1992, p. 83) which – although perhaps (by definition) not a musical ‘style’ per se* –* is recognisable as a practice by its avoidance of pre-agreed formal parameters and emphasis on personal expression and exploring relationships between musical material and co-performers. This practice creates unique situations for improvisors that arise less urgently in genres with fixed musical roles. For example, free improvisors are responsible not just for *what* and *when* they play, but whether their music ‘supports,’ ‘contrasts,’ or ‘ignores’ what others are playing (Golvet et al., forthcoming; Wilson & MacDonald, 2016). They must therefore be sensitive to elements of a situation that, in other styles, might be ‘scaffolded’ onto the tempo or ensemble section.^11^ These additional elements may therefore influence the kinds of affordances and decisions (including whether they are experienced *as* decisions) that are available to free improvisors with respect to other improvising practices. Nevertheless, all of the Lab musicians were also fluent in multiple genres, and their collective free improvisations incorporated a range of ensemble styles and sizes, invoking various relationships to rhythm, tonality, and other parameters.

As Noë (2015) argues, artists themselves perform a type of questioning *within* their own *praxis*. We should take the ‘*experimental*’ in ‘experimental free improvisation’ seriously, in the sense that – by pre-supposing minimal *provisos* – its performance becomes a mode of making those *provisos* that *do* appear more explicit and available for philosophical consideration. For example, although more ‘conventional’ musical material – melodies, for example, or ‘grooves’ – are not explicitly forbidden in free improvising scenes, their presence is not taken for granted (at least among the ensembles we worked with, but see also Wilson & MacDonald, 2016, p. 1036). Thus, when they do arise, their enabling/constraining effect is more pronounced, as well as – by way of contrast – making visible the unspoken norm that such parameters are ‘out of place.’ I therefore suggest that the phenomena and forms of cognition and attention – the feeling of impulses, ‘split attention,’ and deliberate decision-making I describe below – present in free improvisation should also be present in more structured improvisational forms (albeit perhaps with much *more* decision-making ‘scaffolded’ onto more established *provisos*). While this remains an empirical question for further research, I return to this point at the end of the next subsection.

#### Results: Decision-making and agency in improvised music

As mentioned above, a key question for the phenomenology of improvisation is the sense of individual ‘agency’ (understood as wilful, deliberative decision-making) in spontaneous musical decisions. Popular accounts suggest little space for deliberation in expert performance, something Dreyfus (2005, 2007) has extrapolated to argue that ‘thinking’ in general is detrimental to the ‘smooth coping’ of expert performance. Montero (2016, pp. 32–3), on the other hand, criticises this conception as ‘the myth of just-do-it,’ arguing that explicit cognition is not just present but *necessary* for high-level achievement. ‘Cognition-in-action’ here comprises such active capacities as ‘attention,’ ‘conscious control,’ ‘predicting,’ ‘deliberating,’ and ‘self-reflective attention’ (*ibid*, pp. 41–8).

In what follows, I first try to unpick the vague term ‘thinking,’ to discover the ways in which improvising musicians employ active cognition against the background of a *proviso*-structured world. In particular, I articulate the subtle phenomenological distinctions between differing degrees of agency, and how moments of active cognition arise from and fade back into less explicit processes. We shall see that an improvisor’s sense of explicit ownership and control is related to their sense of musical phenomena ‘as’ objects, and that an important part of their skill lies in knowing when and how to more actively assert cognitive control.

While I shall argue for a key role for explicit cognition-in-action, I follow Høffding (2018, p. 107) in taking seriously the strong phenomenological sense of ‘acting without thinking’ that pervades much expert musical performance. It is worth noting that the musicians I worked with repeatedly echoed a Dreyfusian story of active deliberation as disruptive to their improvising process:

Thinking is slower than the action needs to be.

(S6, Violinist)

The optimal situation is that there’s not much thinking, and then it just goes into this state-of-mind... perceiving the environment around me, and then reacting with it and to it.

(S19, Flautist)

If you get to that point of like ‘what should I do here?’... you’re in trouble.

(S8, Saxophonist)

What ‘thinking’ means precisely in this context is of course a central problematic. As many critics of the Dreyfusian ‘automaticity’ account have argued, ‘not-thinking’ while acting (or what we might more formally call ‘pre-reflective’ non-conceptual coping) is not the same as ‘automatic,’ ‘mind-less’ or unconscious behaviour (e.g., Christensen et al., 2016; Zahavi, 2013). And while Dreyfus (2007) highlights coping’s similarities to what Csikszentmihalyi (1987) called ‘flow,’ this latter state appears to be the exception rather than the rule during improvised practice (cf. Høffding, 2018; Montero, 2016), albeit extremely significant when it does happen.

[Group flow] never lasts very long.

(Interviewer): How long are we talking? Like… minutes?

Long would be a minute [laughs] ... But for me, this is the reason why [we're doing] what we're doing. We're trying to make it possible for this to happen.

(S6, Violinist)

Other musicians used ‘flow’ to describe immersion in their individual practice, referring to experiences that Høffding (2018) calls ‘absorbed not-being-there’ (pp. 81–4) or “ex-static absorption” (pp. 84–5). However, the musicians also highlighted how the demands of being a professional ensemble performer require forms of ‘higher order’ and “situational” awareness (Endsley, 1995).

When the flow comes and things just go by themselves, then it's good... If I sit at home, I have no audience, no obligations whatsoever, I can just keep in the flow as long as I want.

But as soon as I'm in a concert situation, or this kind of workshop situation, and there's other people that I have to communicate with, and all this – then I have to have a second layer, which is usually alternating, it can't be at the same time.

There is a need to step out, because there's always a formal frame to this kind of concert... You have to make a step out, and think ‘how much time do I have?’ And ‘what is [my co-performer] doing?’ and – and then jump back in and forget about the rest again.

(V4, Sound artist)

Nevertheless, while coping may indeed involve mindedness, the sense of ‘stepping in and out’ suggests that there remains an important phenomenological difference between what it is like to *cope*, and the experience of *thinking*. It is tempting to equate ‘thinking’ here with its everyday meaning of deliberative, linguistic cognition – with debating with oneself or having an internal monologue over one’s actions. And verbalised thought does indeed play a much larger role in improvising than mythologies of ‘flow’ might suggest. Sometimes it can take the role of attentive deliberation (‘*this sounds weird, maybe I should play low tones instead…*’), but it also frequently takes the form of what Sutton et al. (2011) call “instructional nudges” – verbal cues like ‘*softer*’ or ‘*go – go – faster*’ that guide and trigger less overtly cognitive actions. Or verbalisation can also manifest as ‘mind-wandering’ (cf. Høffding, 2018, p. 77), where a player’s explicit thoughts are completely unrelated to what they are playing (not an ideal state, yet – curiously – often less disruptive than active deliberation).

But while literal verbalisation is not an infrequent occurrence, the experience of ‘thinking’ while improvising resists a simple equation with propositional language. For improvisors, ‘thinking’ refers less to language-use, and more to a state-of-awareness that feels a step removed from action:

I don’t really use words most of the time, but there are clear, conscious, or mental [thoughts]... ‘Now I want to hear *this*’... Or ‘everything is so busy...’

(S20, Pianist)

No words. It’s more like this kind of... It’s really a physical thing. But a very rational physicality.

(V1, Flautist)

It’s not necessarily [verbal], but it’s clear that you are deciding. So I think that in that context, *thinking* means literally deciding something.

(S6, Violinist)

Central to this experience of ‘thinking’ appears to be what we could call an *intentional object* in the classical sense. ‘Object’ here is not necessarily material, but whatever forms the foreground of thought, be that a physical thing, person, or sound, but also something more complex and temporally-stretched – a melody or rhythmic ‘fill,’ for example, or the ‘*this*’ that Pianist S20, above, wants to hear. In this sense, it is easy to see how – if the objects of such ‘thoughts’ were intricately detailed, such as the precise position of one’s finger – such ‘thinking’ would be distracting in just the way ‘automaticist’ thinkers like Dreyfus have argued. Yet as proponents of ‘hybrid’ views – like Christensen et al.’s (2016) ‘meshed cognition’ account, or Bernacer and Murillo’s (2014) account of ‘cognitive enrichment’ – have argued, the habituation of more basic, technical skills provides a framework for focusing attention on higher-order decision-making, yet without closing-off technical skills from conscious control. Which is to say, the elements of such skills, experienced pre-reflectively, always carry the *potential* to become an intentional object, a ‘*this*’ (and tend to do so when things don’t go as expected; cf. Heidegger, 1962, pp. 104–5).

All the same, pre-reflective agency – the intuitive ‘*reacting*’ described by Flautist S19 earlier – retains a phenomenological contrast to this object-focused ‘thinking.’ A key difference between the two modes lies in a sense of distance from the ‘thought-object,’ and the sense of ‘ownership’ of the agent’s interactions with it. Deliberative thoughts appear to come *from* the thinking subject, and to be applied *to* their object; there is something of a ‘gap’ between the thought and any associated action. Pre-reflective ‘reacting,’ by contrast, is more immediate. Ideas – or what free improvisors frequently call *impulses –* are not ‘thought up’ by the performer, but feel like they ‘come *to*’ them, often experienced as a ‘pre-’ or ‘inside’ hearing of a possible sound or musical phrase.

It’s an ‘inside hearing’ of the sound I want to do and it’s – it’s not very conscious, I would say. It’s not like I’m thinking ‘hmm, I’m doing this now.’

(S19, Flautist)

In specific moments I hear things, or there’s things I want to hear.

(S21, Drummer)

These modes of agency are not mutually exclusive. Impulses may arrive spontaneously, but rather than be immediately enacted, become an ‘object’ of thought, to be judiciously inserted into the music at a point that feels appropriate. As one improvisor describes:

It’s not stream-of-consciousness stuff. It’s more like, ‘I have an idea, I’m going to insert it.’ But then there’s a lot of flexibility. And these things can be very spontaneous.

(S14, Bassist)

Yet significantly, this actively- ‘thinking’ consciousness appears to be focused on the broad contours of a decision, rather than the technical details. Bassist S14 later described how, after choosing to introduce a sound with a particularly difficult technique, he distracted himself by thinking of something unrelated to the music in order not to focus on his hands, which he felt would cause mistakes.

Similarly, one of his bandmates described the way he actively *chooses* a moment to add a particular sound. But on starting to play, he is not fully aware of exactly what he is being drawn to do.

I don’t know what will happen when I come in... Of course, when I start, I have an idea of what – I mean, I start with *something*.

(S12, Trombonist)

Or sometimes the impulsive gesture even ‘pre-empts’ the musician’s deliberative interpretation.

I take the instrument in a certain way, that’s usually then I feel like ‘yeah, that’s the one I would have thought of, if I would have thought of it before’ [laughs]

(V2, Cellist)

So far I have identified two phenomenologically-distinct cognitive modes that nevertheless interact during the act of improvising music. These modes are consistent with Christensen et al.’s (2016; 2019) ‘*meshed cognition*’ theory that argues for a ‘hybrid’ between ‘automatic’ and ‘cognitivist’ accounts of expertise. Nevertheless, while cognitive control *does* seem extendable ‘all the way down’ (that is, none of our habitual skills are walled off from conscious interference in the same way that genuinely automatic responses – like sweating or heart-rate – are), it is still the case that improvisors frequently report parallel cases of ‘monitoring’ pre-reflective processes, and even cases of ‘split’ cognitive attention.

‘Monitoring’ describes the experience (often associated with ‘flow’ states or what Høffding (2018, pp. 84-5) calls ‘ex-static absorption’) of ‘watching oneself’ perform. This is an experience of pre-reflectively musicking alongside reflective consideration, something like the simultaneous experience of what Wittgenstein (1958, pp. 66-7) called the ‘I-as-subject’ and ‘I-as-object’ (cf. Legrand, 2007, p. 588). Merleau-Ponty (2012, p. 94) famously described this distinction as we experience it in the ‘double-touch’ phenomenon, where we shift perception of our two touching hands between perspectives of both toucher and touched. What I call ‘monitoring’ suggests that there is no either/or to these perspectives, but both can manifest together, with perhaps one perspective emphasised. In ‘monitoring,’ reflective attention does not rest with the action of playing, but seems to float above it, intervening (if at all) only to change directions with a ‘gist’ or ‘instructional nudge.’

I’m in two places at the same time. I mean, I’m doing a thing. And I’m not... I’m watching [myself] doing a thing.

(V2, Cellist)

That state I talked about, like where I can think about everything, there’s still a distance somehow, like, it’s kind of I have this sensation, almost seeing the hand as not being mine. And it’s not about control... It’s like, it’s allowing it to happen and not controlling it. But there is control at the same time.

(S20, Pianist)

Or musicians may more actively ‘search’ ahead in time, focusing on an affordance they feel approaching in the music and deciding what to do, even while continuing to play their instrument unreflectively in the present moment.

These moments happen a lot – when I feel ‘ah, I lost track [of where I was going].’ And I'm on a track... And I have to find an elegant way to get off, take a turn, and or to fade out, and come back in again. So this is the moment where I'm thinking of the next thing I'm supposed to do.

(V8, Guitarist)

‘Split attention,’ can also describe a more concentrated state, where an improvisor’s monitoring attention is ‘split’ between two (or more) features of the music, either their own playing or others in the group. For example, a guitarist may focus in on the quiet sounds made by the clarinet while also tuning in to the bass, or a drummer may attend simultaneously to two limbs that are playing different rhythms. Yet the sensation of active, deliberative *control* appears to be inversely related to the spread of the player’s attention. For example, deliberately adjusting one limb requires ‘zooming in’ on it, a form of concentration that leaves the others running more on ‘autopilot.’ From the other direction, ‘splitting’ attention requires the agent to take a step back, or ‘zoom out,’ from their own activity, scanning over different elements or hovering ‘back’ above ‘the whole.’

So [my hands] are two, like two parallel instruments that are playing on two tracks. And there are the feet as well, you know? What’s the hi-hat doing? And it’s really fun...

*(Interviewer): Can you concentrate on two tracks at once?...*

I can get the whole picture, if I’m ‘zooming out’ like in a way. Like... hearing everything... But I can’t concentrate on one and the other at the same time. I just get the whole picture. Or I can concentrate on this or that.

(V6, Drummer)

This description supports the association I made above, between deliberative ‘thinking’ and an intentional object. Control and agency appear bound to this ‘object,’ which is foregrounded at the expense of control over other elements of the action. But what comprises the ‘object’ – the ‘*this*’ or ‘*that*’ – has a broad flexibility. It can be a body part or tool – a finger, hand, or drumstick – but can also be a sound itself, or even a run of sounds like a phrase or melody, such that control of the sound presupposes less attention and direct control over the bodily movements that produce it.

Part of the musician’s skill is thus to actively *direct* this attention and control, which she achieves by ‘zooming in’ or ‘out’ on elements of the musical process. The more one zooms out, the more ‘tracks’ one can be aware of, but in surrendering focus, direct control is also ‘given over’ to the process (although it can be skilfully reclaimed at any time). One improvisor used the metaphor of a ‘spotlight’ to describe they way they focused their attention, including the phenomenon of ‘split attention.’

It's a very clear – can be wider or narrower, but it's always just shining on one spot.

*(Interviewer): Do you think you can have two different things in the spotlight? Or do you think the spotlight’s going from, like, from one to the next?*

I think there could be even be several spotlights.

*(Interviewer): Okay.*

And usually one wins. But there can be two or three things at the same time that keep you going.

(V4, Sound artist)

An important observation here is that a ‘spotlight’ of attention always contains just one object, which subsumes other objects as it expands (from ‘finger’ to ‘fingers’ to ‘hand’ to ‘gesture,’ for example). The unusual experience of ‘split attention’ thus shows up less like two objects in a single ‘spotlight,’ and more like multiple spotlights. This goes some way towards explaining how free improvisors make sense of the music in larger ensembles. As ensemble size grows – and especially in experimental contexts where it is difficult to identify one instrument’s (altered or ‘prepared’) sound from another’s – it is not always clear who is playing what or with whom (Goupil et al., 2020, p. 2). While Lab musicians frequently mentioned feeling attuned to one or more others during certain moments, it was not uncommon – especially when the entire ensemble was playing – for this experience to be one-sided; that is, a performer might not recognise that another was responding directly to their material. In moments of collective performance, musicians reported listening to the ‘group sound’ – the way different timbres and textures blended with each other – and either joining it or seeking out ‘gaps’ where their own sound could be distinguished.

I find myself often like, if I hear, maybe, like a particular resonant frequency in the band or a particular pitch, then I feel like, ‘oh, yeah, I might use that pitch.’ And then I might start with that, and – not copy the other person – but maybe, like, latch onto something.

(S14, Bassist)

I often look for gaps. I mean, it's also just the acoustic instrument thing, in more of the sense that, like, if I *don't* ever play in a gap no one will hear me.

(S3, Cellist)

If I want to start something new, I usually do it in, in a frequency gap that's open, that's like, undefined. If I jump on the, on the spot [where] everybody is already doing their thing... it's harder to get heard.

(V4, Sound artist)

In the experience of playing, then, multiple features can stand out as salient, from the musician’s own movements and sounds, to elements of the music itself, including the negative space of ‘gaps’ which afford certain actions. Several of these can co-persist as ‘objectified’ and available for thought, but the more intense the focus, the greater any particular ‘object’ appears.^12^ Cognitive improvisatory skills – including fluent zooming in/out, focusing on particulars, and ‘nudging’ the music in different directions – all presuppose an experience of the music as something holistic and ongoing. And just as much, they presuppose the musical skill of performing without intensive conscious attention, leaving agentive attention ‘free’ to intervene in a self-sustaining process.

This picture therefore suggests two senses in which pre-reflective and deliberative attention combine, giving a sense of the music, while obviously *produced* by the musicians, as nevertheless something external and object-like to the improvising subject-as-agent. Firstly, musical ideas come pre-reflectively to the musician as ‘impulses’ suggested by the situation, which become available as objects of thought. And secondly, deliberative attention can work *with* those musical ideas *as* ideas, while participating in the unfolding music in a pre-reflective way.

In this section, I have given a brief phenomenology of free musical improvisation, arguing that improvisors experience a significant phenomenological difference between active, deliberative ‘thinking,’ and a more immersive, pre-reflective ‘reacting.’ Yet such ‘thinking’ is neither as explicitly verbal nor as disruptive to action as the term ‘deliberative’ might suggest, and my account is more attuned with Christensen et al.’s hybrid ‘meshed’ account of skilled activity rather than Dreyfus’ more dualistic division between coping and mindedness.

I argued that ‘thinking’ is characterised by the experience of an ‘object’ *as* an intentional object of a subject. Such objects need not be material, but include sounds, phrases, melodies, as well as simple or composite physical entities like a string, fretboard, or a guitar. Such ‘thought-objects’ are distanced from the pre-reflective background in the sense of being isolable from their surroundings. The act of isolation is not always under the improvisor’s control; sometimes an affordance will ‘jump out’ at them, sometimes an idea will arrive as an ‘impulse.’ But once present-at-hand for the musician, an object becomes available for deliberation and explicitly chosen action.

This ‘meshed’ mode of coping with diverse objects as they arise likely captures many of our everyday activities. In the flow of musical improvisation, however, they can take different forms, especially in the context of the attentive concentration that accompanies musical immersion. Here, I have noted especially the phenomenon of ‘monitoring,’ where one’s own actions become objects of attention without requiring an assertion of agency. And I have noted ‘split attention,’ where the agent ‘zooms out’ to focus on two (or more) ‘objects’ simultaneously. Yet like monitoring, split attention seems to necessitate a relinquishing of some control in order to widen consciousness, although such control can be reasserted by ‘nudging,’ or by ‘zooming’ back ‘in,’ which again supports a hybrid account of cognitive control over even the most flowing actions.

But to conclude this section, I need to reiterate the caveat I made at its beginning, namely that this phenomenology refers to situated examples from a particular non-idiomatic free improvising scene. This ‘tradition,’ while sonically diverse, is united in attempting to produce music with a minimum of *provisos* by avoiding pre-given parameters of harmony and rhythm, and pushing the material limits of instruments. Furthermore, a significant number of Lab participants, across ensembles, have histories and connections to the so-called ‘Berlin Reductionist’ scene, which typically emphasises the very deliberate placement of sounds, with a high tolerance of tacet-ness that encourages close listening to the discrete timbral and textural relationships that emerge (Blažanović, 2011; Hayward, 2011). This approach arguably allows more space for active deliberation than some ‘busier’ styles of, for example, free jazz improvisation. It also remains an open, empirical question whether the phenomenology outlined in this section accurately encompasses more densely *proviso-*ed improvisational practices, such as Hindustani classical or Scottish traditional folk music.

Nevertheless, I would argue that – in terms of the structural concepts I have outlined here – differences between genres would be a matter of degree rather than kind. Faster, rhythmic musics may be more conducive to ‘flow’-like experiences, and more *provisos* imply more ‘outsourced’ or scaffolded decisions. But this is compatible with an agent ‘monitoring’ and ‘nudging’ their performance in just the way I have outlined here. Furthermore, as I have stressed at several points, free improvisation – while starting with minimal pre-specifications – does not imply a complete *absence* of *provisos.* Rather, by removing conventional parameters, it makes space for implicit *provisos* to become visible – *as provisos –* to both players and audiences. The ‘magnetic’ pull of an emergent rhythm takes on greater salience when it is not taken for granted, creating an imperative for the ensemble to actively join or resist it.

In this way experimental improvisors should be seen as researching or “reorganising” improvisation in Noë’s (2015) sense, laying bare structural concepts that are embedded in the wider *praxis*. While the phenomenology I have outlined remains in an empirical sense provisional, in the next section I develop the concepts further by discussing them in the more general terms of the *modes of knowing* that they imply, which may give a clue to how they might be further applied to other improvising practices in music and beyond.

## *Technē* and *Phronēsis*

So far, I have laid out two complementary ways of looking at improvisation. In Section 2, I gave a 4E-based account of improvisation as a sense-making system, where implicit *provisos* enable and constrain agents to make improvised decisions in response to the dynamic musical ‘object’ that they shape. In Section 3, I explored those decisions from the perspective of the agents themselves, giving a phenomenological account of how reflective consciousness monitors ongoing performance, ‘zooming’ in and out onto different elements, and ‘nudging’ new impulses into action.

Section 3’s findings suggest that many improvised actions are not experienced *as* decisions as such; that there is something about them which is not a musician’s ‘own.’^13^ Section 2 explains this by recognising the improvisor as embedded in a wider socio-material system – a ‘form-of-life’ (Taylor, 1989; Wittgenstein, 2009; cf. MacIntyre, 1981) – that conditions what kinds of musical actions can ‘show up’ as appropriate or even possible. But as I argued in Section 3.1, understanding music-making in systemic terms should not downplay the centrality of individual will and agency. While we are, in a sense, instantiations of a ‘form-of-life,’ we also define ourselves – as *persons* – within and against it, embodying and enacting the (musical and other) values of a community via the development of our own *praxes*. *Praxis* here refers to the kind of projects that we involve ourselves in, that give meaning to our lives and, in a very real sense, constitute who we *are*. As Silverman (2020, p. 5) puts it, “persons emerge… and are enacted because of the dynamical syntheses of our many embodied processes that are in/of our worlds.” Music (like other arts, crafts, and trades) forms a *praxis* because* “*musicing is something worth doing for the sake of the self and others” (*ibid*, p. 8).^14^

*Praxis* therefore goes beyond the technical embodied skills (the *hows*) with which those projects are accomplished, to encompass the *whys* – that is, those skills’ place in a wider form-of-life. This conception, while having roots in Aristotle (1094a10-20),^15^ aligns sympathetically with the 4E program (van der Schyff et al., 2022, p. 198), serving to situate specific musical practices into a fuller, meaning-giving context.^16^ For their part, free improvisors have long emphasised the social and ethical dimensions of their practice (Beins et al., 2011; MacDonald & Wilson, 2020), and a praxial approach has also become increasingly influential in music pedagogy (Elliott & Silverman, 2015).

With its sense of ‘inhabiting’ a musical ‘form-of-life,’ *praxis* therefore offers a link between systemic and individual perspectives, and between the levels of explicit decision-making and implicit *proviso*-navigation, that preserves the role of creative individual agency even in the absence of deliberation. Indeed, as I will argue below, *praxis* suggests a way towards understanding the un-deliberative sense of ‘free agency’ involved in improvisation since, for Aristotle, *praxis* is governed by a ‘mode of knowing’ he calls *phronēsis* (‘practical wisdom’) – a form of situated practical rationality – in ways that parallel how creative production (*poiēsis*) is governed by *technē* or ‘technical skill.’ Both modes draw deeply on habituation, being responses to reasons that are embodied and experienced as *feelings of solicitation* eliciting their enactment.

*Technē* and *phronēsis* are two of the intellectual *aretai*^17^ (*aretai dianoētikai* (Aristotle, 1139b15)), or what I have been calling ‘modes of knowing.’ They are linked by their concern with ‘things that change’ (i.e., unfolding, pragmatic judgements), as opposed to other intellectual *aretai* like *epistēmē* and *sophia* (‘theoretical knowledge’ and ‘wisdom’), which have to do with universals (or, in more contemporary terms, with propositional or conceptual content) (Aristotle, 1139a5-10). They differ, as mentioned above, in that *technē* is expressed in *poiēsis* (‘making’ or ‘production’); it has a definite object, whose meaning (including whether it ‘works’ or not) comes from how it fits into our broader ‘form-of-life.’ *Phronēsis*, meanwhile, is concerned with *praxis*, ‘ethical action’ in the broad sense of referring to our entire ‘form-of-life’ as such, encompassing our sense of the ‘good’ which in turn shapes the value of all of our other acts.

The relevance of *technē*, as a mode of knowledge, to musical performance should be self-evident, describing as it does practical skills such as mastery of one’s instrument and the ability to enact musical knowledge (e.g., using the instrument to play specific sounds or scales). This ‘musico-technical expertise’ (as Høffding and Schiavio (2021, p. 822) call it) relies on tacit, embodied, habits but, as described above, is not unconscious or automatic in virtue of this. Musical *technē* is knowledge enacted in a situation-specific way – how hard to push the piano keys, for example, or how long to let the sustain linger – and is in this sense non-propositional, since it cannot be captured in a set of rules. An under-emphasised point about *technē* is that – as a non-propositional form of knowledge – we only express it *in the action itself*. That is, I only really ‘have’ my *technē* of playing the guitar when I am holding the guitar and playing it. The skill is not like a line of computer code in my brain, waiting to be executed, but the enactment of a *disposition* (*hexis*) to react in certain, sense-making ways when I pick up the instrument and play it (Aristotle, 1040a10-15).

*Phronēsis*, as a form of expertise, works analogously as a form of situation-specific ethical judgement. Its possessor – the *phronimos* – is often described as one who sees the *right* thing to do in a particular situation, where what is ‘right’ might not conform to any universal(isable) moral law, and which he might arrive at without rational deliberation. An important point is that its acquisition, like that of *technē*, also relies on developing a *hexis* or disposition to act that is refined through practice. But even more than *technē*, it is a product of enculturation: as Gallagher (2007, p. 206) puts it, *phronēsis* can’t be taught, but is acquired by ‘hanging around with the right people’ – and then doing as they do. Which is to say, *phronēsis* is a sensitivity to the values of a community of ethical experts, developed through immersive practice.

This intangibility distinguishes *phronēsis* from *technē* (Aristotle 1040b20-25). While *phronēsis* likewise comprises a disposition to act, it is not aimed at a ‘product’ (a house, a piece of music) but at the entire *praxis* or form-of-life itself. Unlike *technē*, *phronēsis.*

is not *solely* a disposition accompanied by reason [*hexis meta logou*], a sign of which is that it is possible to forget such a disposition but not to forget *phronēsis*.

(Aristotle 1140b25-30, my emphasis)

That *phronēsis* ‘cannot be forgotten’ suggests a form of cultural embodiment that goes even deeper than the habituated dispositions of technical skills. *Technē* – rooted in bodily habits – is certainly difficult to forget, if we consider the muscle-memory that develops with expertise on an instrument (though we do indeed become ‘rusty’ or ‘lose our chops’ without ongoing practice). But Aristotle’s distinction suggests something stronger, that I would argue we can make sense of by considering his later (1142a25-30) likening of *phronēsis* to perception (*aisthēsis*) rather than (propositional) knowledge (*epistēmē*).

To take a musical example, a jazz pianist *immediately* hears when she (or her bandmates) hit an out-of-key note. Although there is a *reason* (and a history) for *why* that note sounds ‘off,’ her perception is not *reasoned* – it just sounds wrong. But the converse is also true – a melody or phrase that is *in* key will always sound that way. Even if the pianist learns non-western scales or experiments with atonal music, she will not be able to ‘unhear’ the ‘natural’ sense made by a scale she is trained in. She has been initiated into a ‘form-of-life’ – the *praxis* of a jazz musician – that structures her perception of the phenomena around her. However, *phronēsis* – as a virtue – encompasses much more than this perception. It is concerned with the very action itself and the question of ‘what to do.’ Hearing an off-key note from a bandmate, an expert improvisor might intuitively see a subtle response, ‘rescuing’ them from embarrassment, perhaps through the ‘old jazz musician’s trick’ of repeating an error to make it look deliberate.

It might be argued that *phronēsis –* strictly-defined as an *ethical* capacity – is not directly applicable to the practice of musical improvisation described here. But the simple example above shows the kinds of aesthetic (playing in key) and social (avoiding embarrassment) values that are at stake in an improvisation. For Aristotle, too, ‘ethics’ was a much broader topic than what is today discussed as morality. *Hexis* (disposition) is etymologically-related to both *ēthos* (habit) and *ethos* (character) – who one *is* (one’s ‘form-of-life’) is inseparable from what one *does* (one’s *praxis*) (Aristotle 1103a-1103b). Our values are both inherited from our community (things sound ‘good’ or ‘right’) and asserted in our actions (the ‘right’ thing to do).

This interrelation of disposition, habit, and character reflects what Carlisle (2014, p. 27), following Ravaisson, calls the ‘double law of habit,’ the phenomenon by which the more an action is repeated, the less its performance is actively *felt*. Noting the Aristotelian origins of this idea, Carlisle (*ibid*, pp. 23–6) draws upon the metaphor of a pathway, emphasising especially its temporality in both directions: not only does enacting a disposition reflect one’s past experience, but in being enacted, the disposition *itself* suggests the future course (that is to say, it offers a ‘scaffold’ for the sense-making decision of which it is an ongoing part). But a crucial point is that the disposition or habit must in each case be *enacted*. The concept of *hexis* suggests not a fixed reflex or program, but a *tendency.* Like *technē*, a *hexis* is only actualised in the context of a situation, where it leads potential actions – what ‘makes sense’ or is ‘right’ to do – out of past experience.

Of course, in an unfolding improvised performance, there will be countless possibilities of what ‘the right thing’ could be within the *provisos* of a given genre or style. While the value of any aesthetic decision will contain a high degree of subjectivity, some choices will nevertheless be judged ‘better’ than others – they will make ‘more sense’ to the community at large, be more ‘appropriate’ in the context of a particular concert or cultural ‘scene’ (cf. Wilson & MacDonald, 2017).

Crucially, what makes a ‘good’ musical choice is also in unfolding flux. To perform an identical musical act, even in a similar situation, might have a very different meaning. Many – if not most – improvising styles place a high premium on novelty and creativity. (Post)modern ‘free’ improvisation, as we have seen, often takes this to an extreme, rejecting traditional concepts of rhythm and tonality altogether. Yet while critiques can be made of this Modernist emphasis on ‘the new,’ it is also true that other improvising traditions (including folk musics and non-western classical music) also encourage innovations within the form, prizing personal creativity over blind imitation (Nettl, 2016, pp. 175–7; Bailey, 1992, pp. 8, 17). Improvisors are therefore incentivised to recognise their ‘typical’ or habitual responses to affordances, and in some experimental modern styles, the chief *proviso* could even be said to be avoiding anything that might appear ‘cliché.’ Across genres, both musicians and audiences positively distinguish between mere displays of technical skill – however expert – and the kind of innovative responsive expertise that is experienced as great art. That is to say, there is an important distinction between being a mere *virtuoso*, and being musically *virtuous* in a *phronetic* sense (cf. van der Schyff et al., 2022, p. 198).

At the very least, then, we can say that a *phronēsis-like* form of *responsive expertise* is as central to successful improvisation as technical mastery (cf. McDowell, 1994, p. 84).^18^ That is to say, *technē* – the skill of directly and un-deliberatively producing and adjusting the desired sounds from one’s instrument – is a necessary but not sufficient ability for the act of improvisation. One must also be able to expertly choose both *what* and *when* to play. And this requires a sensitivity to precisely what would *make sense* in a particular situation – a sensitivity to all of the structural *provisos* listed earlier (*key, rhythm, and so forth*) as well as particular social dynamics, such as who has been playing what already (*are one’s co-performers dominating or reticent?*), the mood of the audience (*are they bored or digging it?*), and an awareness of one’s own practical limitations (*will your contribution be heard? How will it fit in the greater context?*).

But as I argued in Section 3, this sensitivity does not imply an improvisor is actively *thinking* about all of these considerations. Like technical *provisos*, social *provisos* do not need to be the objects of explicit deliberation, but rather form the *background* against which a musician responds to their situation. Insofar as they are conscious, it is through the ‘situational awareness’ that Christensen et al., (2016, p. 43) argue “often occurs without explicit inferential reasoning processes.” As McDowell (2007) argues*,* although the *phronimos* may not arrive at a logical conclusion, nor even be aware of or able to articulate precisely the reasons *why* she performs a certain action in a certain context, this does not mean her act is not ‘responsive to reasons.’ Rather, she *feels* that her actions *make sense* in the context of both the specific activity and her overall form-of-life.

*Phronēsis* – being a social rather than instrumental expertise – is felt less as a bodily solicitation and more as an *intuition*. Like an improvisor’s ‘impulse,’ it is felt as ‘coming *to*’ the performer, a consciousness of an opportunity to do something that would make sense, with a greater or lesser degree of imperative attached. That the ensuing act itself becomes conscious is by no means given. Many of our cultural dispositions – the comfortable distance we should stand from a conversation partner, for example – are enacted without ever entering our attention. In a similar way, I would suggest that a musician’s ‘responsive expertise’ is likewise enacted by-and-large pre-reflectively – at least when she is ‘at home’ in the situation. In the performance mindset, a musician is situationally-attuned to the musico-social *provisos –* those unspoken yet powerfully normative expectations that shape what opportunities may manifest.

Yet at the same time, we saw in Section 3 that affordances may enter the improvisor’s attention as an intuition without being immediately acted upon – that a musician may consciously choose to enact or restrain the afforded ‘impulse.’ In an interestingly reflexive way, a musician may then develop the skill of responding to her own intuitions, developing an increasing sensitivity to the affordances of each unique musical situation. Much as I argued that *technē* can only be actualised when the musician is holding her instrument, so *phronēsis* (or its musical equivalent) only exists *between* the agent and the ethical (or aesthetic) situation. Our *praxis* comprises a dialectic between our habits and dispositions on the one hand, and the situation on the other, mediated by *phronēsis* which – as a ‘rational disposition but not *merely* so’ (Aristotle 1140b25-30) – intervenes on the process to align it with our aesthetic/ethical values.

We therefore return again to our initial paradox. While musical improvisation appears rooted in the habits of *technē* and a *phronēsis*-like responsive expertise that is centred on *praxis*, the latter also includes acquiring a sensitivity to one’s typical reactions. The ‘monitoring consciousness’ we saw in Section 3 suggests how such habits are recognised and halted during the ‘flow’ of performance. We saw a clear phenomenological contrast between object-focused cognitive experience, and immersed motor-intentional ‘coping.’ Yet we have seen that these two systems are intimately meshed, not just in the sense that they operate in parallel, but in how ‘objects’ (i.e., phenomena experienced *as* objects) emerge and melt back into one from the other. A musician may deliberately seek out sonic objects as she feels her way through the music, or they may autonomously ‘intrude’ as *impulses,* as if from ‘outside.’ In these cases, the improvisor’s skill lies in choosing whether, when, and how to enact these impulses in a way that makes sense in the context – that is, in ways that align with the values of her musical ‘form-of-life’ (be that *novelty*, or *fidelity*, or *accuracy*, or *expressiveness*, or some combination of these and more).

But the term ‘choosing’ – with its everyday, deliberative sense – may overstate the explicit agency at work here. The enactment of an ‘impulse’ is most frequently not an explicit consideration of different options, but an adjustment to what *feels* right, what *makes sense* within the constraints of the *provisos*. An expert’s possibilities for action are experienced as ‘external’ – as *potentiae –* and explicit cognitive control may extend to little more than a ‘nudge,’ introducing a musical element that is immediately and reflexively responded to as a solicitation within one’s own playing, and amongst the unfolding, dynamic situation. Within that situation, the musician is immersed in a sea of affordances – sounds, *provisos*, and social dynamics – that become more and less salient from moment to moment as she makes sense of and *with* the music.

## Conclusion

In this paper, I sought to unpick the apparent paradox that improvised music uses its grounding in individual and cultural habits to spontaneously create something *new*. I offered three, interconnecting perspectives, each of which suggests how individual decision-making is embedded in a broader, socio-material ‘form-of-life,’ that is itself interpreted by an agent in her personal *praxis*. I first argued that musical improvisation is a form of ‘participatory sense-making’ in which coupled agents form a *system* with an emergent, autonomous musical ‘object,’ structured by *provisos* that – as both enablers and constraints – lay out decision pathways. I then offered an *individual* perspective of navigating those pathways, based on phenomenological fieldwork with practicing improvisors, where I articulated how explicit attention monitors, deliberates, and intervenes on ‘objectified’ musical phenomena and on the musician’s own pre-reflective responses. Finally, I brought these perspectives together under an Aristotelian framework, arguing that the Philosopher’s concepts of *technē* and *phronēsis* help us make sense of rational, pre-reflective decision-making, and their roots in dispositions (*hexeis*) that are not fixed, but enacted *between* the agent and the world.

These perspectives are complementary, and together suggest ways of thinking about agency, decision-making, and creativity as distributed processes that blur the boundaries between subject and object, individual and community, without erasing them. Two important points follow as a result. Firstly: how *provisos*, background awareness, and a culturally-ingrained sense-of-rightness all act as *constraints* on what can be meaningfully performed by an improvisor – indeed, of what can even ‘show up’ as a possibility for deliberation. But secondly, these structures also *enable* particular possibilities *to* make sense. The particular sounds that a musician chooses to play – even in the non-deliberative ‘flow’ – are guided by a normative sense of ‘rightness’ that precedes her, and which she embodies in virtue of being initiated into a particular (sub)culture.

This cultural, *praxial* dimension means that musical decisions are therefore *ethical* decisions, in two interconnected ways. Firstly, because their ‘rightness’ suggests they are particular instantiations of generic communal values (like ‘good,’ ‘beautiful,’ or ‘interesting’), which are therefore asserted as worthwhile; and secondly because they are a matter of *decision*. Even insofar as the guiding values precede the performer, she is always in the position of asserting them anew. The improvisor’s skill of monitoring her pre-reflective performances is thus seen as the skill of recognising, refraining or adjusting her reactions in order to (musically) enact a particular instantiation of ‘*the good*.’

This further skill of becoming *aware* of one’s habits – of intervening, altering, or avoiding them – opens a space to explore different possibilities. Our dispositions are not fixed, but reaffirmed and reshaped every time we enact them. What makes sense must still be negotiated with our community, but one of the values of art is that it creates a space where such possibilities can be explored and developed – or in Noë’s (2015) terms, *reorganised*. And if we view the artistic space as a microcosm of our extended, cultural ‘form-of-life,’ then the skills of intervening, questioning, and developing that we find in improvised art have a relevance to how we approach the constraints and possibilities that shape our broader social world.

## Data Availability

In line with privacy considerations, the research data (interviews) will not be stored in a public repository.

## Footnotes

1 I agree with Small (1998) that *music* should be understood first and foremost as something we *do*.

2 Thanks to an anonymous reviewer for this illustrative example.

3 On ‘forms-of-life’ see Taylor (2016; cf. 1989); cf. Wittgenstein (2009). MacIntyre (1981, p. 190) argues that artistic *praxis* reveals and is guided by the goods of a “certain kind of life”; and Sparshott (1988, p. 141) refers to art as a ‘*Lebensform*’ (a ‘form of life’) in Wittgenstein’s sense.

4 The term ‘*proviso*’ in this sense was coined by Maya Hey, and initially developed in conversation with her.

5 See Lewis (1996).

6 See Blažanović (2011).

7 If she plays the traditional pentatonic (plus a ‘blue’ note).

8 In Gibson’s (1979, pp. 127–9) sense.

9 ‘Object’ does not quite do the job in this context, as the experienced phenomenon is more like an ongoing process or shared activity. But as we will see in Section 3, what can be ‘objectified’ by an experiencing subject go far beyond material entities.

10 The exceptions were almost entirely due to age. Four members of one ensemble were aged ≤ 25 years at the time of the Lab. However, all four are also professional musicians and recent graduates of a well-regarded, practice-based university jazz program.

11 Although see Beins (2007) for an example of how these choices can be fixed as *provisos* in an improvisational context).

12 Something which likely relates to the availability of working memory – see Baars & Franklin (2003, pp. 167–8).

13 For more on ‘own-edness’ and expert performance, see Bergamin (2017, p. 420).

14 Note that ‘musicing’ in this praxial sense (cf. Elliott, 1995) differs from Small’s (1998) ‘musicking.’ See van der Schyff et al., (2022, p. 18).

15 All citations from Aristotle are from the *Nicomachean Ethics* and referred to by Bekker number. I have relied on Bartlett & Collins’ (2011) translation, referring to Rackham (1926) for Greek text.

16 This is not a coincidence. 4E theories owe a great deal to phenomenology which, especially in its Heideggerian formulation, is in many senses a development of Aristotelian ideas (see Sheehan, 1975; Kisiel, 1993; Bowler, 2008). Together with an engagement with virtue ethics (MacIntyre, 1981; McMullin, 2019; Delacroix, 2022), these form strands of what is becoming a comprehensive neo-Aristotelian conception of human life and action.

17 ‘Excellences’ or ‘virtues.’ However, although the *aretai* have an important ethical dimension, ‘virtue’ here cannot be understood in terms of simplistic ‘good/bad’ morality. Aristotle himself (1097b25–30) uses the example of a flute-player to illustrate ‘virtue’ in the broader praxial sense that I discuss here.

18 Høffding & Schiavio (2021, p. 122) discuss this as “interactive expertise” (not to be confused with Collins’ (2010) ‘interactional expertise’), although the ‘responsiveness’ I discuss here refers to the musician’s interaction not just with their fellow players, but with the musical *event* itself.
